# The influence of endocrine disruptors on the gut microbiota

**DOI:** 10.55730/1300-0144.6124

**Published:** 2025-10-13

**Authors:** Kader UĞUR

**Affiliations:** Department of Endocrinology and Metabolism, Faculty of Medicine, Fırat University, Elazığ, Turkiye

**Keywords:** Endocrine disruptors, gut, microbiota, human health

## Abstract

Endocrine disruptors (EDs) are closely associated with the second brain, the microbiota-derived enteric nervous system, commonly referred to as the gut microbiota. The microbiota plays a crucial role in human health and the development of diseases. In today’s industrialized world, the presence of EDs in air, water, and soil leads to primary human exposure through dermal contact and ingestion. The impact of these EDs on the microbiota remains unclear. EDs that disrupt the balance of the gut microbiota may contribute to a range of disorders, including metabolic (obesity, diabetes mellitus), cardiovascular (vascular stenosis, cerebrovascular disease), reproductive (infertility, ovarian and testicular tumors), neurological (dysfunction of the amygdala, cortex, and cerebellum), and behavioral disorders (dementia, depression, anxiety, and schizophrenia). This review examines the effects of commonly encountered environmental EDs on the gut microbiota and summarizes the most recent findings on this topic. The concept of the microbiota-derived enteric nervous system and the modulation of the hormonal system through interactions between microorganisms and environmental chemicals have prompted specialists in endocrinology and metabolism to reconsider patient management and treatment strategies. This necessitates a comprehensive evaluation of treatment options that incorporate microbiome data. The information presented in this review will help illuminate future research directions and serve as a valuable resource for subsequent studies.

## 1. Introduction

Exposure to endocrine disruptors (EDs) is inevitable in modern industrialized societies, as they are ubiquitous in the environment through air, water, and soil. These substances commonly enter the human body through inhalation, dermal absorption, or ingestion. The challenges we increasingly face involve chemical-dependent agriculture and food production, as well as the accumulation of electronic waste, plastics, and other synthetic materials. Once inside the body, EDs can interfere with the production, release, transportation, and function of hormones by exerting either agonistic or antagonistic effects [1]. Numerous epidemiological and experimental studies have demonstrated that EDs disrupt the balance of the gut microbiota, leading to a variety of health disorders, including metabolic, cardiovascular, reproductive, neurological, and behavioral diseases [2]. Among various sources of EDs, chemicals present in agricultural and food products pose a greater risk than those originating from industrial sources. Despite an expanding body of evidence indicating the influence of EDs on the microbiota, definitive conclusions remain limited. Dr. Ugur’s [3] research provides detailed information on EDs, including commonly encountered compounds such as bisphenol A (BPA), pesticides, polychlorinated biphenyls (PCBs), phthalates, phytoestrogens, parabens, heavy metals, triclosan (TCS), and trichloroethanes (Table). This review primarily focuses on the effects of these substances on the microbiota-derived enteric nervous system, particularly the gut microbiota.

## 2. Microbiota

The gut microbiota plays a vital role in maintaining human health, as most of its microorganisms are either beneficial or commensal to the host. This microbiota provides several essential benefits, including protection against infections, facilitation of nutrient and energy absorption from food, and enhancement of immune system function [4,5]. Research suggests a potential link between the gut and the brain, arising from correlations in gut bacterial composition. Studies utilizing germ-free mouse models indicate that a healthy microbiota is essential for normal hippocampal development and neuronal morphology, both of which are critical for optimal brain function [6]. The human microbiota consists of bacteria, fungi, archaea, and viruses, with the human microbiome representing the collective genomes of these microorganisms [7]. These microorganisms are primarily acquired from environmental exposure and dietary sources. In early infancy, the gut microbiota is dominated by *Lactobacillus* and *Bifidobacterium*, whereas *Firmicutes* and *Bacteroides* become more abundant in later stages of life. In addition to sex-related differences, microbial diversity is a critical factor in promoting healthy aging. Research indicates that the gut microbiota influences brain function and mood, leading to the identification of gut–microbiota-associated pathways as the “second brain” [8] An imbalance in the gut microbiota, known as dysbiosis,, may result from direct bacterial interactions or their metabolites, leading to increased intestinal permeability and the production of hormone-like substances [2,9].

The interaction between EDs and the gut microbiota remains an emerging topic of considerable scientific interest.

## 3. Endocrine disruptors and the gut microbiota

### 3.1. Bisphenol-A (BPA)

BPA is an endocrine disruptor widely used in the manufacturing of various plastic products and epoxy resins. BPA-containing products are characterized by their clarity and rigidity, which distinguish them from BPA-free alternatives. As a result, BPA is commonly incorporated into the production of various items such as water bottles, sports equipment, CDs, and DVDs [9,10]. Studies investigating the effects of EDs on the gut microbiota have specifically examined exposure to BPA and ethinyl estradiol (EE) in both female and male monogamous and biparental mice. Using 16S rRNA sequencing on bacterial DNA extracted from fecal samples, analyses were conducted separately for each sex. Exposure to BPA or EE has been associated with an increased abundance of certain bacterial taxa, including *Bacteroides*, *Mollicutes, Prevotellaceae, Erysipelotrichaceae, Akkermansia, Methanobrevibacter*, and *Sutterella*, which are linked to disorders such as inflammatory bowel disease, metabolic syndrome, and colorectal cancer. Conversely, some females exposed to BPA and EE exhibited an increased abundance of the beneficial bacterium *Bifidobacterium* in their fecal samples. Therefore, it is suggested that BPA and EE may disrupt the gut microbiota and induce systemic effects [2]. Furthermore, BPA-induced alterations in the gut microbiota indicate dysbiosis [10–12], which may contribute to the development of type 1 diabetes mellitus (T1DM). T1DM is an autoimmune disease characterized by the destruction of pancreatic β-cells, brought about by proinflammatory immune cell infiltrates and cytokines/chemokines [13]. Apart from genetic predisposition, environmental exposures seem to play a significant role in the increasing incidence of T1DM [14,15]. The involvement of the gut microbiota in T1DM progression is noteworthy, as it promotes the development and function of immune cells, while immunity, in turn, regulates the composition of the gut microbiota [16]. Additionally, the release of nonrecyclable BPA through microplastics and nanoplastics may cause various adverse effects, including physical injury, reduced growth rates, behavioral alterations, disturbances in lipid metabolism, gut microbiota dysbiosis, impaired intestinal barrier function, neurotoxicity, reproductive damage, oxidative stress, and immune dysfunction [17].

### 3.2. Pesticides

Pesticides are widely used in various agricultural settings, including fields, vineyards, and gardens, to enhance crop yields, particularly in response to increasing population density in recent years [18]. However, pesticide use has led to environmental contamination and adverse effects on various organisms. Dichlorodiphenyltrichloroethane (DDT) is an estrogen-mimicking endocrine disruptor (ED) with lipophilic properties, enabling it to easily penetrate cell membranes and accumulate in adipose tissues and organs such as the adrenal glands, testes, thyroid, liver, and kidneys for extended periods. Perinatal exposure to DDT has been associated with an increased risk of obesity and impaired glucose tolerance [19]. Organophosphates have also been shown to exert detrimental effects on the metabolic system, with low-dose exposure contributing to the development of metabolic disorders such as obesity and diabetes mellitus [20]. Furthermore, chronic dietary exposure to chlorpyrifos, an organophosphate pesticide, has been shown to promote weight gain, increase food intake, and decrease insulin sensitivity by altering the gut microbiota in mice [21].

### 3.3. Polychlorinated biphenyls (PCBs)

PCBs are a group of EDs that accumulate in adipose tissue and have been widely used as insulating materials in electrical equipment. Their presence has been associated with an increased incidence of type 2 diabetes mellitus [22]. Early-life exposure to neurotoxic substances such as PCBs can adversely affect neuronal development by disrupting the interaction between the host and its microbiota. Imbalances in the gut microbiota can lead to the production of metabolites and neuroactive peptides that disrupt gut–brain communication, resulting in behavioral changes such as depression, anxiety, mood disturbances, cognitive alterations, and increased stress sensitivity. These neurotoxic compounds can increase intestinal permeability and the expression of proinflammatory cytokines, rendering the host more susceptible to colonization by pathogenic bacteria and their toxins [23]. Mouse studies have shown that oral exposure to PCBs increases intestinal permeability and elevates levels of proinflammatory cytokines in intestinal tissues [24]. PCB exposure also alters the composition of the gut microbiota, decreasing the abundance of *Bacteroides* and *Proteobacteria* while increasing *Actinobacteria, Verrucomicrobia*, and *Firmicutes* [25]. Previous research has suggested that an increase in the *Bacteroidetes/Firmicutes* ratio in the gut is related to systemic inflammation [26]. Alterations in microbiota diversity can result in an increased abundance of *Fusobacteria* [27]. Furthermore, in vitro studies have shown that reduced expression of tight junction proteins is associated with increased intestinal permeability [28]. Essentially, the microbiota contributes to both the development and degeneration of neurons. Exposure to PCBs can significantly alter the gut microbiota, potentially contributing to the onset of various diseases.

### 3.4. Phthalates

Phthalates, also known as phthalate esters, are widely used to increase the flexibility and durability of plastics. These compounds are present in a wide variety of products, including personal care items such as shampoos, lotions, nail polishes, and cosmetics, as well as toys, candles, detergents, medical equipment, pharmaceutical coatings, building materials, flooring, wallpapers, paints, and adhesives. They are also known to contribute to the characteristic new car odor. Exposure to phthalates has been associated with several health conditions, including overweight, obesity, insulin resistance, diabetes mellitus, and cardiovascular disease [29]. Numerous animal and human studies have emphasized the roles of maternal diet, gut microbiota, and ingestion of contaminated food in increasing the risk of obesity [30]. Research has demonstrated that oral exposure to specific phthalates alters the *Firmicutes*-to-*Bacteroidetes* ratio and changes the abundance of certain bacterial species, including *Akkermansia* and *Prevotella*, leading to disruptions in lipid metabolism and reproductive dysfunction. Moreover, exposure to di-(2-ethylhexyl) phthalate has been linked to alterations in gut microbial metabolites, contributing to disturbances in cholesterol homeostasis and neurodevelopmental disorders. Previous studies have indicated that supplementation with probiotics capable of regulating gut health—such as *Lactococcus* or *Lactobacillus* species—may help prevent phthalate-induced hepatotoxicity or testicular toxicity by modulating host gene expression, regulating gut bacteria, and facilitating the fecal elimination of phthalates [31].

### 3.5. Phytoestrogens

Phytoestrogens are plant-derived compounds that mimic 17β-estradiol and exert estrogenic activity. The use of estrogen therapy remains controversial due to its potential effects on breast cancer, endometrial cancer, and cardiovascular disease, as reported by the Women’s Health Initiative [32]. The “estrobolome,” a collection of bacterial genes within the gut microbiota, plays a crucial role in regulating sex hormone homeostasis in humans. Lignans have been associated with *Clostridium methoxybenzovorans*, *Firmicutes*, and *Bacteroidetes*, whereas isoflavones are linked to *Faecalibacterium prausnitzii*, *Lactobacillus*, and *Enterococcus*. These microorganisms within the gut microbiota are commonly associated with phytoestrogen metabolism. Studies suggest that elevated estrogen exposure may influence the development, progression, and treatment outcomes of hormone-dependent cancers [33]. The effects of phytoestrogens on human health—particularly among individuals with breast cancer—may be modulated by their metabolism, which is, in turn, influenced by the host microbiota in both the small and large intestines. Despite extensive research, whether phytoestrogens are beneficial or detrimental to human health remains uncertain. This uncertainty arises from interindividual variability, as the effects depend on factors such as age, health status, and the composition of the gut microbiota [34].

### 3.6. Parabens

Parabens are a class of endocrine disruptors widely used as preservatives in pharmaceutical and cosmetic products to extend their shelf life. These compounds and their corresponding salts are extensively used in various industries owing to their potent antibacterial and antifungal properties. They are mainly found in personal care products such as shampoos, conditioners, cosmetics, facial cleansers, and skincare formulations [35]. Recent studies have demonstrated an association between exposure to cosmetic contaminants such as parabens and neurotoxicity, leading to cognitive impairment and neurodevelopmental disturbances. With the ongoing acceleration of industrialization and urbanization, the demand for cosmetics, pharmaceuticals, and personal care products has markedly increased. Consequently, the continuous release of persistent organic pollutants into aquatic ecosystems has raised serious concerns regarding their effects on biodiversity and environmental health [36]. Disruption of gut microbiota homeostasis can cause disturbances in lipid metabolism, a key factor contributing to the onset of nonalcoholic fatty liver disease (NAFLD). Despite current evidence, the precise relationship between dietary factors and gut microbiota homeostasis in NAFLD development remains poorly understood. Furthermore, a murine study investigating the toxicity of food additives such as methylparaben and ethylparaben under different dietary conditions demonstrated that chronic paraben exposure exacerbates NAFLD via microbiota-dependent arachidonic acid metabolism pathways [37,38].

### 3.7. Heavy metals

Exposure to common environmental contaminants such as arsenic, nanoparticles, and lead has been shown to alter the gut microbiota in rodent models. Research indicates that perinatal lead exposure in mice induces alterations in the gut microbiota, which are associated with body weight changes, particularly in males [39–41]. Arsenic contamination in drinking water has been associated with the development of cancer and various other diseases. However, the exact pathophysiological mechanisms underlying chronic arsenic exposure and its impact on the gut microbiota remain poorly understood. A murine study investigating the effects of arsenic on the gut microbiota and host metabolism revealed that alterations in colonic bacterial composition—particularly in *Bacteroidetes* and *Firmicutes*—were both time- and concentration-dependent in response to arsenic exposure. Additionally, arsenic exposure resulted in alterations in nitrite and nitrate levels in the colon and liver, accompanied by disruptions in nitrogen metabolism. These findings suggest that arsenic exposure affects both the microbiome and microbiome–host nitrogen metabolism, potentially contributing to the development of pathogenic phenotypes [42].

### 3.8. Triclosan (TCS)

Triclosan (TCS), an antimicrobial agent, is widely present in household and personal care products, being incorporated into more than 2000 consumer items. EDs detected in surface waters and sediment layers pose significant ecological risks by disrupting the structure and function of aquatic ecosystems. A study reported that carbanilide antibacterials such as triclocarban and TCS are widespread environmental contaminants, prompting calls to ban consumer products containing these agents [43]. Another study demonstrated that TCS exposure increases colonic inflammation and exacerbates the progression of colitis-associated colorectal tumors, with implications for antibiotic resistance and alterations in the human microbiome [44]. Despite these concerns, a study investigating TCS exposure in mothers and infants revealed an increase in *Proteobacteria* abundance in stool and urine samples, with minimal impact on the overall gut microbial community. These findings highlight the necessity of rigorous consumer safety assessments for antimicrobial personal care products to evaluate their effects on the human microbiome and the emergence of antibiotic resistance [45].

## 4. Conclusion

The use of EDs has become increasingly widespread and poses a substantial risk to human health. Although some EDs may be incorporated into products for purposes such as food preservation, they directly influence the gut microbiota. EDs disrupt the homeostasis of the gut microbiota, thereby interfering with tissue, thyroid, and pancreatic hormone regulation, leading to multiple physiological dysfunctions. Further research is essential to elucidate the complex interactions between the cumulative effects of dietary contaminants and alterations in diet and lifestyle. Additionally, further studies are required to validate the effectiveness of primary prevention strategies aimed at mitigating the growing obesity epidemic driven by environmental pollutants and unhealthy lifestyle factors. The present study provides a comprehensive overview of the detrimental effects of EDs on biological systems, underscoring the urgent need for in-depth research on these compounds to safeguard human health.

## Figures and Tables

**Table:** Common endocrine disruptors of the gut microbiome and their detailed functions [3].

Endocrine disrupting chemicals	Potential effects
Bisphenol-A (BPA)	Triggering obesity and diabetes
Pesticides	Hormonal changes (Insulin, TSH, etc.)
Polychlorinated biphenyls (PCBs)	Hormonal changes (Insulin, TSH, etc.)
Phthalates	Disruption of hormone synthesis and degradation
Phytoestrogens	Disruption of hormone synthesis and degradation
Parabens	Hormonal changes
Heavy metals	Inflammation, intestinal tumors, etc.
Triclosan (TCS)	Inflammation and tumors

This list of endocrine disruptors has been redrawn with minor modifications [3].
